# Caractéristiques épidémiologiques, cliniques, thérapeutiques et évolutives des patients atteints de glioblastome cérébral: série de cas pris en charge au centre d´oncohématologie du Centre Hospitalier Universitaire Mohammed VI de Marrakech en 2016 et 2017

**DOI:** 10.11604/pamj.2021.39.191.28298

**Published:** 2021-07-08

**Authors:** Moulay Yassine Belghali, Saadia Ba-M´hamed, Brahim Admou, Maroua Brahimi, Mouna Khouchani

**Affiliations:** 1 Laboratoire de Recherche Morpho-Science, Faculté de Médecine et de Pharmacie, Université Cadi Ayyad, Marrakech, Maroc,; 2 Laboratoire de Pharmacologie, Neurobiologie, Anthropologie et Environnement, Université Cadi Ayyad, Marrakech, Maroc,; 3 Laboratoire d´Immunologie, Centre de Recherche Clinique, Centre Hospitalier Universitaire Mohammed VI, Marrakech, Maroc,; 4 Laboratoire de Recherche B2S, Université Cadi Ayyad, Marrakech, Maroc,; 5 Laboratoire d´Anatomie Pathologique, Hôpital Mohammed V, Safi, Maroc

**Keywords:** Glioblastome, épidémiologie, chimiothérapie, radiothérapie, IDH, *wild-type*, mutant, centre d’oncohématologie, cancer, *performance-status*, Glioblastoma, epidemiology, chemotherapy, radiotherapy, IDH, wild-type, mutant, Department of Hematology-Oncology, cancer, performance-status

## Abstract

Le glioblastome est la tumeur cérébrale maligne primaire la plus commune. Malgré les avancées diagnostico-thérapeutiques, il continue d´être de mauvais pronostic. Nous avons étudié une série de cas pour décrire les caractéristiques épidémiologiques, cliniques, thérapeutiques et évolutives des patients atteints de glioblastome admis en 2016 et 2017 au centre d´oncohématologie (COH) à Marrakech. Il s´agit d´une série de cas rétrospective uni-centrique. Analyse documentaire des données épidémiologiques, cliniques, radiologiques, anatomopathologiques, thérapeutiques et évolutives de 40 patients. Le glioblastome représentait 47,6% des tumeurs intracrâniennes traitées. L´âge moyen des patients est 52,4±12,3 ans. L´impotence fonctionnelle et les signes d´hypertension intracrânienne dominaient les symptômes. Les tumeurs étaient préférentiellement pariétales (44%) et de grande taille (57,5%). L´aphasie était liée à la taille tumorale (p=0,042). Neuf cas étaient des glioblastomes-IDH1-wildet un cas de glioblastome-IDH1-mutant. La médiocrité de l´indice Performance-status à l´admission au COH était corrélée au délai prolongé entre la chirurgie et l´admission au COH (p= 0,034). Les patients présentant des déficits sensitifs étaient plus âgés (62,5±3 ans) que ceux qui n´en avaient pas (51,2±12 ans) (p=0,045). Les femmes hospitalisées ont bénéficié de chimioradiothérapie précocement (1,5±1 mois) que les hommes (2,3±1,2 mois) (p=0,03). La survie était de 13,6±5,3 mois sans lien ni au délai chirurgical (p=0,076), ni au délai d´admission au COH (p=0,058) ni au délai du début de la chimioradiothérapie (p=0,073). Les caractéristiques épidémiologiques, cliniques, radiologiques et évolutives de notre échantillon sont comparables à la littérature. Le profil moléculaire n´est pas systématiquement réalisé. Malgré des délais de prise en charge élevés, aucun lien à la survie n´était décelé.

## Abstract (en)

Glioblastoma is the most common primary malignant brain tumour. Despite advances in diagnostic and therapeutic treatments, it is still associated with poor outcome The purpose of this study of cases is to describe the epidemiological, clinical, therapeutic and evolutionary features of patients with glioblastoma admitted to the Department of Hematology-Oncology (DHO) in Marrakech in 2016 and 2017. We conducted a literature review of epidemiological, clinical, radiological, anatomopathological, therapeutic and evolutionary data from 40 patients. Glioblastoma accounted for 47.6% of treated intracranial tumours. The average age of patients was 52.4±12.3 years. Functional impotence and signs of intracranial hypertension were the main symptoms. Tumours mainly occurred in the parietal region (44%) and were large (57.5%). Aphasia was related to tumour size (p=0.042). Nine cases had glioblastomas-IDH1-wild and one case had glioblastoma-IDH1-mutant. On admission, patients had poor performance-status. This was due to a prolonged time between surgery and DHO admission (p= 0.034). Patients with sensory impairments were older (62.5±3 years) than those without sensory impairments (51.2±12 years) (p=0,045). In-patient women received chemoradiotherapy (1.5±1 month) earlier than men (2.3±1.2 months) (p=0.03). Survival was 13.6±5.3 months; it was unrelated to the time to surgery (p=0.076), the time to DHO (p=0.058), and the time to chemoradiotherapy (p=0.073). The epidemiological, clinical, radiological and evolutionary features of our sample were comparable to literature data. The molecular profiling was not systematically realized. Despite prolonged treatment times, no link to survival was detected.

## Introduction

Le glioblastome (GBM) est la tumeur cérébrale maligne primaire la plus commune, représentant jusqu´à 80% des cancers cérébraux et ceux du système nerveux central (SNC) [1-3]. La médiane d´âge des patients ayant un GBM est approximativement 64 ans. Néanmoins, il peut survenir à n'importe quel âge, même chez l´enfant [1]. Son incidence est légèrement plus élevée chez l´homme avec un sexe-ratio de 1,6/1 et chez les Caucasiens par rapport à d'autres groupes ethniques [3]. Le GBM est un cancer de mauvais pronostic. De plus, l'âge avancé, le bas indice de l´OMS *Performance Status* (PS) et la résection incomplète sont les principaux facteurs pronostiques péjoratifs [4]. Chez les patients âgés, la survie médiane est inférieure à quatre mois avec des soins palliatifs seuls [5]. Cependant, les caractéristiques moléculaires, telles que la mutation de l'isocitrate déshydrogénase 1 et 2 (IDH-1 et de l´IDH-2) et la méthylation du promoteur MGMT, confèrent un pronostic plus favorable [6, 7]. Les patients se présentent souvent avec un tableau d´hypertension intracrânienne (HTIC) (céphalées et déficits neurologiques focaux d´évolution progressive). Les convulsions sont présentes chez 25% de patients et peuvent arriver à un stade plus avancé de la maladie chez 50% de patients [8]. La TDM montre une masse tumorale avec, éventuellement, un effet de masse marqué et un œdème environnant et parfois de l´hémorragie. L´IRM, quant à elle, montre des signes de nécrose et d´hémorragie intratumorale et d'œdème vasogénique [9].

En postopératoire, la chimiothérapie à base de Temozolomide concomitante de radiothérapie (CRC) suivies par une chimiothérapie adjuvante est le protocole thérapeutique le plus couramment pratiqué. Des traitements plus récents comprennent, entre autres, l'anti-angiogenèse (Bevacizumab) et l'immunothérapie. Chez les personnes de 70 ans ou moins, le protocole standard Stupp est habituel. Chez les personnes âgées, la radiothérapie est généralement administrée sous forme de traitement plus court, mais même dans ce contexte, l'ajout de Temozolomide augmente la survie, en particulier dans les tumeurs qui présentent un promoteur de MGMT méthylé [10]. Les progrès thérapeutiques ont amélioré la survie médiane à plus de 15 mois chez les patients sous traitement [11, 12]. Dans l'ensemble, le taux de survie relative à 1 an était de 41,4% pour les patients diagnostiqués aux États-Unis entre 2000 et 2014, avec une amélioration de 34,4% à 44,6% pour les périodes de 2000 à 2004 et de 2005 à 2014, respectivement. Malgré ces améliorations progressives des taux de survie à court terme au fil du temps, le taux de survie à 5 ans est resté relativement constant, avec un taux de survie de seulement 5,8% à 5 ans après le diagnostic [13], alors que la progression de la maladie chez 70% des patients ayant un GBM se fait en moins d´un an après le diagnostic [14, 15]

La connaissance des différentes caractéristiques de ces patients est susceptible de garantir une bonne gestion des approches thérapeutiques et de leur procurer une meilleure prise en charge [16]. C´est dans cet esprit où s´inscrit cette étude qui a pour but de décrire les caractéristiques épidémiologiques, cliniques, thérapeutiques et évolutives des patients ayant un GBM cérébral admis au centre d´oncohématologie (COH) du centre hospitalier universitaire en 2016 et 2017.

## Méthodes

**Conception de l´étude:** il s´agit d´une série de cas rétrospective uni-centrique. La collecte des données a bénéficié de l´approbation du comité d´éthique de la faculté de médecine et de pharmacie de Marrakech.

**Participants:** les patients inclus sont ceux pris en charge au COH du 01/01/2016 au 31/12/2017 et dont le diagnostic de GBM a été confirmé par l´examen anatomopathologique. D´autre part, seuls les patients dont le dossier médical est présent à l´archive du COH ont été recrutés.

**Sources de données:** c´est une étude documentaire du dossier médical de chaque patient.

**Taille de l´étude:** le nombre de cas admis au cours de la période d'étude a déterminé la taille de l'échantillon.

**Variables:** la collecte de données figurant sur les dossiers médicaux est faite par une grille exploitant lesdites caractéristiques. Notamment, les caractéristiques épidémiologiques, cliniques, thérapeutiques et évolutives des patients ayant un GBM cérébral. D´autre part, nous avons calculé: *le délai-consultation:* temps écoulé entre le début des symptômes et la première consultation; *le délai-chirurgie:* temps enregistré entre le début des symptômes et l´intervention chirurgicale; *le délai-COH:* temps écoulé entre le début des symptômes et l´admission au COH; *le délai-CRC:* temps écoulé entre le début des symptômes et la date de la première séance de CRC; *le délai-chirurgie-COH:* temps passé entre la chirurgie et l´admission au COH; *le délai-COH-CRC:* temps entre l´admission au COH et le début de la CRC; *Le délai-Chirurgie-CRC:* temps entre la chirurgie et la date de la première séance de CRC et *la survie:* temps écoulé entre le début de la symptomatologie et le décès du patient.

**Biais:** concernant les données manquantes, il y avait lieu d´omettre les cas concernés et d´analyser les données restantes.

**Méthodes statistiques:** l'analyse descriptive a consisté au calcul des fréquences absolues et relatives pour les variables qualitatives, et des paramètres de positionnement et de dispersion pour les variables quantitatives (moyenne, écart-type). La distribution normale des variables est étudiée par le test de Kolmogorov-Smirnov. La comparaison des variables qualitatives a fait appel au test statistique de Chi2 de Pearson et celui de Fisher si nécessaire. Le test de Mann Whitney et celui de Kruskal Wallis sont utilisés pour comparer les variables continues. Le seuil de significativité est retenu pour un p < 0,05.

## Résultats

**Caractéristiques épidémiologiques de la population:** nous avons colligé 40 cas de GBM (10 en 2016 et 30 en 2017) parmi 84 cas de tumeurs cérébrales pris en charge au COH. Cela représente 47,6% des processus tumoraux intracrâniens pris en charge et 0,94% (0,48% en 2016 et 1,37% en 2017) des cancers pris en charge au COH, tous types confondus. La moyenne d´âge des patients était de 52,4±12,3 ans. La tranche d´âge la plus touchée était (50, 60 ans). Le sexe masculin a prédominé avec un sex-ratio de 1,66. 60% des patients proviennent des régions sud du pays. Un seul patient n´avait pas de couverture médicale, cinq patients en avaient une couverture privée et 34 patients (85%) bénéficiaient de la couverture médicale nationale.

**Caractéristiques cliniques de la population:** après le début des premiers symptômes, les patients ont mis en moyenne 3,1±3,1 mois pour consulter avec une différence non significative entre hommes et femmes (p = 0,184) (2,4±2,3 mois et 4,2±4,1 mois respectivement). A l´interrogatoire, 74% des patients n´avaient pas d´antécédent personnel, médical, ou chirurgical. Le reste des patients avaient de l´hypertension artérielle ou du diabète. Un seul cas a rapporté un cas de tumeur cérébrale chez son frère. L´impotence fonctionnelle (hémiparésies, hémiplégies, paresthésies et/ou aphasie) était le principal signe clinique motivant la consultation (92,5%) suivie par les signes d´HTIC (céphalées et vomissements) (70% des patients), les troubles visuels (strabisme, photophobie et cécité progressive) (17,5% des patients), les troubles de conscience (12,5% des patients), le syndrome cérébelleux (principalement un vertige) (15% des patients) et les convulsions qui ont été rapportées par 20% des patients. Une tuméfaction frontale droite avec adénopathies cervicales droites ont été rapportées par un seul patient. En préopératoire, cinq patients avaient un PS = 0, treize patients avaient un PS = 1, huit patients avaient un PS = 2, huit présentaient un PS = 3 et six patients avaient un PS = 4. Le tableau clinique des patients était dominé par une altération de l´état général, de la conscience et de la vision, des troubles moteurs ainsi que des signes d´HTIC. Nous avons constaté que plus la tumeur était volumineuse, plus les patients souffraient d´aphasie (p=0,042) en préopératoire et plus ils souffraient de céphalées, sans signification statistique (p=0,086).

**Caractéristiques para cliniques de la population:** en préopératoire, la tomodensitométrie (TDM) a été réalisée chez 80% des patients et l´imagerie par résonnance magnétique (IRM) chez 57,5%. Ces deux examens ont permis de mettre en évidence le processus tumoral et son siège. A part un seul cas de tumeur du tronc cérébral, toutes les tumeurs de notre série étaient de localisation sus-tentorielle. Les tumeurs chevauchaient entre les différents lobes cérébraux et leurs localisations étaient préférentiellement pariétales, frontale et temporale dans 44%, 23% et 15% des cas. D´autre part, sept cas présentaient une tumeur de petite taille (<10/10/10mm), dix cas présentaient une tumeur de taille moyenne (entre 20/20/20 à 30/30/30mm) et 23 cas avaient une tumeur de grande taille (30/30/30 à 85/85/85mm). L´étude anatomopathologique du matériel d'exérèse (75% des cas) ou des biopsies (15% des cas) a suivi la classification et les recommandations de l´OMS de l´an 2007 dans 75% (30 cas) (concluant sur un diagnostic anatomopathologique de GBM) ou de l´an 2016 dans le reste des cas (concluant sur un diagnostic anatomopathologique de GBM IDH1-*wild* dans neuf cas et un GBM IDH1-*mutant* dans un seul cas).

**Caractéristiques de prise en charge chirurgicale des patients:** le délai-chirurgie dans notre série était de 2,8±2,9 mois (allant de 8 jours à 381 jours) et n´influençait pas la survie (p=0,076, r = 0,300). La chirurgie a consisté en une exérèse large dans 72,5%; une exérèse partielle dans 12,5% et une biopsie chez 15% des cas. Nous n´avons pas pu avoir d´information concernant les modalités chirurgicales adoptées.

**Caractéristiques évolutives de la population:** à leur admission au COH, 1,4±1 mois après la chirurgie, l´examen clinique des patients a révélé un déficit moteur (hémiparésies et hémiplégies) chez 55%, un trouble de langage chez 20%, une altération de l´état général chez 15%, une altération de la conscience (syndrome frontal, obnubilation, désorientation spatiale et convulsions) chez 12,5%, des troubles visuels (strabisme convergeant gauche, strabisme bilatéral, nystagmus vertical et nystagmus horizontal) chez 7,5%, des paresthésies chez 10% et des adénopathies chez un seul patient. L´examen des patients a aussi montré que les ceux présentant des déficits sensitifs étaient plus âgés (62,5 ans en moyenne) que ceux qui ne l´étaient pas (51,2 ans en moyenne) (p=0,045). Les patients qui ont présenté des troubles de conscience en post-opératoire étaient plus âgés (54 ans en moyenne) que ceux qui ne les ont pas présentés (41,4 ans en moyenne) (p=0,057). De même pour les troubles du langage, qui ont été présentés par des patients plus âgés (54 ans en moyenne) que chez les plus jeunes (46 ans en moyenne) (p=0,084). D´autre part, nous avons noté des récidives radiologiquement évoquées chez 30% des patients opérés et que, plus la tumeur était volumineuse, plus les patients souffraient de récidives en post-opératoire (p=0,095). Le PS à l´admission au COH était de 0, 1, 2 et 3 chez 7,5%; 37,5%; 42,5% et 12,5% des cas respectivement. A part une corrélation avec le délai *chirurgie-COH* (p=0,034), ce score n´avait pas de lien avec les autres délais mesurés. En fin, la survie était de 13,6±5,3 mois. Les hommes survivant moins que les femmes (12,6±3,9 et 15,4±6,9 respectivement) (p=0,193).

**Caractéristiques de prise en charge thérapeutique de la population:** le délai entre le début de la symptomatologie et l´admission au COH était de 4,2±3,1 mois, avec une différence non significative entre hommes (3,6±2,3 mois) et femmes (5,2±4,1 mois) (p = 0,246). Le traitement médical prescrit, aussi bien, en postopératoire immédiat qu´après admission au COH, se basait sur les antalgiques, les corticoïdes, les antiépileptiques, les diurétiques osmotiques et les antiacides gastriques. Au COH, 37 patients ont bénéficié d´une chimio-radiothérapie concomitante (CRC) 2,1±1,3 mois après y avoir été admis, deux patients ont été récusés et un patient a reçu sa radiothérapie à l´Institut National d´Oncologie. A noter que les femmes hospitalisées ont bénéficié de CRC plus précocement (1,5±1mois) que les hommes (2,3±1,2 mois) (p=0,03). Le protocole de radiothérapie consistait en l´administration d´une dose de 60Gy sur 6 semaines à raison de 5 séances par semaine, en fraction de 2Gy/séance. Et la chimiothérapie consistait à l´administration du Témodal* (Temozolomide) à la dose de 75 mg/m2 par jour. Suivie de 6 cycles supplémentaires du même médicament (150 à 200 mg/m2 aux jours 1 à 5 de la semaine tous les 28 jours) pendant six mois avec surveillance du bilan biologique. A noter un délai entre le début de la symptomatologie et le début de la CRC s´élevant à 6,2±3,6 mois et qui était, à son tour, plus court chez les hommes (5,8±3 mois) que les femmes (6,7±4,6 mois) sans pour autant que cette différence soit statistiquement significative (p=0,845). Par ailleurs, à part des relations proches à la signification statistique (*survie/délai-chirurgie, survie/délai-COH et survie/délai-CRC*, p=0,076; p=0,058 et p=0,073 respectivement) nous n´avons pas noté de lien entre la survie et les délais mesurés (Tableau 1).

**Tableau 1:** corrélations entre les différents délais de prise en charge des patients de notre série et leur survie

	Délai-consultation	Délai-chirurgie	Délai-COH	Délai-CRC	Délai-chirurgie-COH	Délai-COH-CRC	Délai-chirurgie-CRC
**Moyenne**	3,1±3,1	2,8±2,9	4,2±3,1	6,2±3,7	1,4±1,0	2,1±1,3	3,4±1,8
**corrélation de Pearson (avec la survie)**	0,000	0,300	0,319	0,303	0,169	0,014	0,158
**Valeur asymptotique bilatérale**	p = 1,000	p = 0,076*	p = 0,058*	p = 0,073*	p = 0,325	p = 0,934	p = 0,359

Une tendance proche de la signification statistique pour lien entre une survie plus prolongée aux délai-chirurgie, délai-COH et délai-CRC élevés

## Discussion

Caractéristiques de la population et déterminants de santé: dans notre série, le GBM représente 47,6% des processus tumoraux intracrâniens pris en charge dans notre hôpital. Chatel *et al*. en a déjà décrit une incidence de 7 à 9/100.000/an [17] alors que le rapport de l´an 2013 du *Central Brain Tumor Registry of the United States* a décrit une incidence de 3,19/100000 habitants avec une tendance vers l´augmentation compte tenu du vieillissement de la population et du développement et généralisation des moyens de diagnostic [18, 19]. Les GBM sont généralement observés dans la région supratentorielle mais très rarement trouvés dans le cervelet et la moelle épinière en concordance avec nos résultats [20]. De même que dans notre échantillon, la moyenne d´âge était de 55,5 ans et la tranche la plus touchée fut [51 ans, 60 ans (dans une étude menée en 2008 au niveau du même établissement [21]). Le GBM est principalement diagnostiqué chez les adultes avec un âge médian de découverte de 64 ans. L'incidence augmente avec l'âge avec un pic entre 40-84 ans et une baisse après 85 ans [19]. Concernant l´enfant, la prévalence de 1,25% que nous avons notée (1 cas sur 40, en deux ans) avoisine celle trouvée dans le même contexte en 2008 [21] et correspond aux données de la littérature statuant sur sa rareté chez l´enfant [19]. La prédominance masculine que nous avons notée était plus marquée dans le même contexte en 2008 (sex-ratio = 3) [21]. L´avènement du régime national d´aide médicale et les efforts entrepris pour sa généralisation peuvent expliquer cette amélioration entre 2008 et 2017 lui permettant de rejoindre l´incidence observée dans d´autres contextes (sex-ratio = 1,57) [19].

Symptomatologie: nos patients ont présenté un tableau clinique fait de maux de tête, de convulsions, de déficits neurologiques focaux, de syndrome cérébelleux, de vision floue, d´altération de l'état de conscience et d´autres symptômes associés à l´augmentation de la pression intracrânienne tels que des nausées-vomissements. Cela représente le tableau clinique typique des tumeurs cérébrales [22]. Nos patients ont mis du retard pour bénéficier de la première consultation pour leurs symptômes. En effet, l´évolutivité rapide du GBM fait que les malades qui en souffrent consultent en moins de 2 mois à 3 mois [23, 24]. A noter que lors de l´admission au COH, l´examen physique des patients a montré que les patients présentant des déficits sensitifs étaient plus âgés (62,5 ans en moyenne) que ceux qui n´en présentaient pas (51,2 ans en moyenne) (p=0,045). Ces symptômes dépendent de l'emplacement et de la taille de la tumeur et sont similaires aux symptômes produits par tout processus expansif cérébral. Ils dépendent également de l´importance de l'œdème cérébral, caractéristique du GBM et cause majeure de morbi-mortalité en raison de l´augmentation de la pression intracrânienne, source d´ischémie, d´engagement cérébral et de décès [25].

**Bilan radiologique:** la TDM a été réalisée chez 80% de nos patients. Elle met en évidence un processus tumoral généralement de grande taille [26] (23/40 grandes tumeurs dans notre échantillon). A la TDM, on observe des marges irrégulièrement épaisses, un centre hypodense et irrégulier représentant une nécrose, un effet de masse marqué et un œdème environnant. L'hémorragie est parfois observée, la calcification est rare et un rehaussement irrégulier et hétérogène intense des bords est presque toujours présent [9]. Les tumeurs de notre série se situaient préférentiellement dans la région pariétale, frontale et temporale, alors que généralement elles sont décrites être localisées au niveau du lobe frontal, temporal, pariétal, occipital (40%, 29%, 14%, 3% respectivement) et dans les structures plus profondes du cerveau dans 14% des cas. Cependant, une hétérogénéité considérable est décrite dans la distribution anatomique des gliomes cérébraux [27]. Nos résultats confirment cette hétérogénéité de localisation.

A l´IRM, réalisée chez 57,5% de nos patients, on observe en T1, une masse hypo à iso-intense au sein de la substance blanche accompagnée d'un signal hétérogène central (nécrose, hémorragie intra tumorale). Après injection du Gadolinium, on observe un rehaussement variable mais quasi systématique, typiquement périphérique et irrégulier avec des composants nodulaires et entoure généralement la nécrose. La captation du produit de contraste est une caractéristique essentielle du GBM. Elle témoigne de l´angiogenèse tumorale et a été notée chez tous nos patients. Au T2/FLAIR, on observe un aspect hyper intense entouré d'œdème vasogénique. Des vides d'écoulement sont parfois observés [9]. Dans notre série, l´effet de masse fut hautement associé à la taille tumorale (p=0,0001) et à l´aphasie (p=0,025). L´œdème cérébral est une caractéristique quasi systématique, il a été observé dans 90% des patients, l´effet de masse qui en résulte a été noté dans 72,5% des cas. L´envergure de l´œdème peritumoral et la nécrose sont prédicateurs de mauvais pronostic du GBM. Ces caractéristiques sont facilement reconnaissables sur l´IRM en postopératoire et pourraient guider la prise en charge thérapeutique [28].

**Prise en charge chirurgicale:** le délai chirurgical était, à son tour, élevé. En effet, en cas de suspicion de tumeur gliale de grade III ou IV, le délai entre le premier examen neuroradiologique diagnostique et l´exérèse chirurgicale ou la biopsie doit être le plus court possible (21 jours maximum) [29]. Une résection totale macroscopique est généralement recommandée (72,5% dans notre population) si possible. Cependant, la résection chirurgicale complète du GBM est impossible car les cellules cancéreuses migrent activement le long des structures cérébrales [30]. Des analyses rétrospectives ont indiqué que la résection totale macroscopique peut améliorer les résultats de survie, même chez les patients âgés [31]. Nous avons constaté que plus la tumeur fut volumineuse plus les patients souffraient de céphalées, d´aphasie en préopératoire et plus ils présentaient des troubles moteurs ce qui est attribué à l´augmentation de pression intracrânienne consécutive [28]. Nous avons noté des récidives radiologiquement évoquées chez 30% des patients opérés. La taille tumorale initiale est un facteur pronostique important dans la récidive du GBM [32]. De surcroit, la capacité des cellules du GBM à migrer et à envahir les tissus cérébraux sains est remarquable. Cette invasion cellulaire comprend quatre étapes de base: le détachement des cellules invasives de la masse tumorale primaire, l'adhésion à la matrice extracellulaire (ECM), la dégradation de la matrice et la migration cellulaire [33].

**Classification anatomopathologique:** dans notre échantillon le profil IDH1 n´a été examiné que dans 25% des cas. Sur le plan histologique, le GBM est caractérisé par une forte densité cellulaire, une haute activité mitotique, des atypies cytonucléaires marquées, de vastes zones de nécrose et une prolifération endothéliocapillaire caractéristique [34]. La révision de l´an 2016 de la classification de l'Organisation Mondiale de la Santé des tumeurs du SNC a restructuré la classification des gliomes, principalement avec l'incorporation de caractéristiques moléculaires en plus de l'aspect histopathologique [35]. Pour le diagnostic du GBM, la détermination du statut de mutation IDH a été incluse, ce qui a donné lieu à des sous-groupes distincts, à savoir le GBM à IDH-sauvage et le GBM à IDH-mutant [35, 36]. Paradoxalement, la recherche du marquage Ki-67 et p53 ne figure pas dans les recommandations de l´OMS et est, pourtant, demandée chez 23,3% des cas. Les marquages immunohistochimies Ki-67 et p53 augmentent avec le grade du gliome. Une valeur Ki-67 correspondant à huit mitoses par champ de microscope est décrite comme *cut-off value* séparant les gliomes de grade II et III avec 93,7% de spécificité et 75% de sensibilité [2]. Pourtant, le diagnostic histologique du GBM (grade IV) est simple et ne souffre pas de discordances entre lames et entre anatomopathologistes [35]. Dans le GBM de type IDH-sauvage, il existe également plusieurs variantes histologiques. En l´occurrence, les GBMs à cellules géantes, les gliosarcomes et le GBM épithélioïde [10, 37]. Toutefois, les recommandations de traitement ne diffèrent pas en fonction de la variante histologique [38]. Le GBM de type IDH-sauvage correspond au GBM primaire d´évolution clinique plus agressive se développant *de novo*. Il représente 90% des patients atteints de GBM [36]. À l'inverse, le GBM à IDH-mutant qui est généralement secondaire à un gliome de bas grade représente environ 10% des patients avec généralement un meilleur pronostic. En plus du statut de mutation IDH, il existe d'autres changements génétiques et épigénétiques caractérisant ces 2 sous-groupes [35, 36]. La classification 2016 de l'OMS a également ajouté un nouveau sous-type sous les gliomes de grade IV, en l´occurrence: gliomes H3F3A ou HIST1H3B/C K27M (H3-K27M)-mutants et gliomes médians diffus [10]. Ils surviennent principalement chez les enfants et les jeunes adultes et sont caractérisés par un sombre pronostic [39]. Ces tumeurs ont déjà été classées comme GBMs, mais sont maintenant considérées comme une entité distincte.

**Management post-chirurgical:** nos patients ont enregistré un grand retard pour bénéficier de CRC postopératoire. En effet, le délai entre la chirurgie et la CRC doit être compris entre 4 et 6 semaines pour obtenir des résultats optimaux [40]. Après chirurgie, à part un seul cas, nos patients ont tous bénéficié d´un suivi thérapeutique fait de chimio-radiothérapie standard de première intention correspondant au protocole défini par Stupp [41]. Pour les patients avec un GBM nouvellement diagnostiqué, âgés de moins de 65 ans avec un bon indice *Kornofsky Performance Status*, la dose optimale est de 60 Gy sur 6 semaines à raison de 5 séances par semaine, en fraction de 2Gy/séance [42]. Aucun bénéfice n´a été prouvé avec des doses supérieures [43, 44], ni avec des régimes hyperfractionnés ou avec l´utilisation de radio-sensibilisants [45].

Le profile MGMT ne fut demandé chez aucun de nos patients. Le bénéfice du Temozolomide peut être largement attribuable aux patients qui ont une méthylation du promoteur MGMT qui fait taire épigénétiquement le gène, la MGMT étant cruciale dans l'activité de réparation de l'ADN, ce qui entraîne une résistance au traitement par Temozolomide [6]. Malgré les retards enregistrés par les femmes tout au long de la prise en charge depuis le début de la symptomatologie, une fois admises au COH, ces dernières ont bénéficié de CRC plus précocement que les hommes. Les déterminants culturels et économiques de santé dans le contexte local pourraient être la cause de cette disparité. Six de nos patients ont été vus aux urgences pour une dégradation de leur état général et ont reçu des traitements symptomatiques. Les toxicités courantes du temozolomide comprennent les nausées et la myélosuppression, en particulier la thrombocytopénie et la neutropénie. Ils surviennent plus fréquemment pendant la période du traitement adjuvant [6]. Aucun bénéfice n'a été démontré avec des schémas thérapeutiques plus longs ou à forte dose de Temozolomide. Des doses élevées sont associées à une plus grande toxicité et à une détérioration de la qualité de vie [46, 47].

Par ailleurs, nous avons noté que plus la tumeur était volumineuse, plus il y avait des récidives en post-opératoire (p=0,095). L'ajout d'un traitement antiangiogénique avec le Bevacizumab, un anticorps monoclonal humanisé qui inhibe le VEGF, a été étudié dans 2 grands essais randomisés. Malgré l'allongement de la survie sans progression dans les deux essais, le Bevacizumab était associé à une toxicité accrue, alors qu'il n'y avait pas de différence de survie globale [48, 49]. Les options de traitement dans le contexte de rechute ou de récidive sont moins bien définies. En effet, une proportion importante de patients est non éligible pour un traitement de deuxième intention. Les options comprennent une résection chirurgicale supplémentaire, une ré-irradiation, des thérapies systémiques telles que la Lomustine ou le Bevacizumab, des approches combinées ou des soins de soutien seuls [50, 51].

Une tendance proche à la signification statistique a montré que la survie était plus *prolongée lorsque le délai-chirurgie, délai-COH et délai-CRC étaient élevés* (Tableau 1). Ces résultats sont paradoxaux, puisqu´on s´attendait à des résultats inverses. Cela est peut être attribué aux complications chirurgicales, aux complications liées à la CRC ou à une insuffisance des soins palliatifs qui ont fait de sorte à ce que les patients qui bénéficient précocement de chirurgie et de CRC décèdent en premier.

**Corticothérapie:** nos patients ont tous bénéficié d´un traitement symptomatique à base de corticoïdes. Cela est fait dans un objectif de lutte contre l´œdème cérébral en restaurant la barrière hémato-encéphalique [52]. L'œdème cérébral et l'inflammation induits par le GBM sont largement traités avec de la Dexaméthasone, un glucocorticoïde synthétique. Cependant, une étude récente a rapporté que cette molécule n'avait aucun effet significatif sur le volume de l'œdème peritumoral chez la majorité des patients recrutés [53]. En outre, de nombreuses études ont rapporté qu´elle inhibe la prolifération et la migration des cellules GBM. Néanmoins, de récents résultats contradictoires ont remis en question la certitude largement acceptée concernant la corticothérapie pour le GBM [54].

**Limites de l´étude:** ce travail présente quelques limites à la généralisation de ses déductions dont l´aspect rétrospectif et documentaire de collecte de données susceptible de faire perdre des informations importantes mais ne figurant pas sur les dossiers des patients. D´autre part, nous n´avions pas pu avoir assez de données concernant la prise en charge chirurgicale. De plus, les deux années étudiées ne nous ont pas permis de colliger un nombre plus important de patients qui aurait pu faire aboutir à davantage de liens entre les variables étudiées.

## Conclusion

Les GBM restent des tumeurs très agressives, accompagnées d'un éventail de complications liées à la maladie sous-jacente et au traitement. Il faut noter que l´absence d´un registre local du cancer, le niveau d´accessibilité financière, culturelle et géographique aux services de santé et la présence d´autres centres d´oncologie (publiques et privés), ne nous permet pas de statuer sur la réelle incidence locale du GBM. Actuellement, l'approche multimodale du GBM reste la pierre angulaire de l'approche thérapeutique dans le contexte nouvellement diagnostiqué. Mais l'utilisation des nouvelles techniques chirurgicales faciliterait les résections tout en minimisant la morbidité chirurgicale. Le profilage moléculaire des GBM est à même de permettre l´identification de nouvelles options thérapeutiques. L´amélioration de l´accessibilité au soin, la réduction des délais de prise en charge, la reconnaissance et la prise en charge des symptômes, combinées à des soins palliatifs sont susceptibles d´améliorer la qualité de vie des patients.

### Etat des connaissances sur le sujet

- L'âge avancé, le mauvais score performance-status et la résection incomplète sont des facteurs pronostiques négatifs pour le glioblastome.

### Contribution de notre étude à la connaissance

- L´incidence des cas de GBM pris en charge au COH de Marrakech a augmenté durant la période d´étude, les délais de consultation et de prises en charge sont allongés par rapport aux délais conseillés.
- La survie n´a présenté aucun lien aux délais des prises en charge;
- Une disparité liée au genre concernant l´accès au soin a été constatée.
